# Effects of Heat Treatment Procedures and Diamond Burnishing on Tensile Properties and Surface Integrity of Additively Manufactured 17-4PH Steel Cylindrical Parts

**DOI:** 10.3390/ma19112192

**Published:** 2026-05-22

**Authors:** Galya Duncheva, Jordan Maximov, Vladimir Dunchev, Angel Anchev, Vladimir Todorov, Yaroslav Argirov, Kalin Anastasov, Hristian Mitev

**Affiliations:** 1 Department of Material Science and Mechanics of Materials, Technical University of Gabrovo, 5300 Gabrovo, Bulgaria; jordanmaximov@gmail.com (J.M.); v.dunchev@tugab.bg (V.D.); anchev@tugab.bg (A.A.); v_todorov@tugab.bg (V.T.); 2 Center of Competence “Smart Mechatronic, Eco-and Energy-Saving Systems and Technologies”, Technical University of Gabrovo, 5300 Gabrovo, Bulgaria; kalinanastasov@abv.bg (K.A.); hmitev@tugab.bg (H.M.); 3 Department of Material Sciences, Technical University of Varna, 9010 Varna, Bulgaria; jaroslav.1955@abv.bg; 4 Department of Industrial Technologies and Design, Technical University of Gabrovo, 5300 Gabrovo, Bulgaria

**Keywords:** combined post-processing concept, laser powder bed fusion, 17-4PH stainless steel, heat treatment, diamond burnishing, tensile properties, surface integrity

## Abstract

This article presents a new combined post-processing concept to improve the quality of laser powder bed fusion (LPBF) of 17-4PH stainless steel (SS) cylindrical parts fabricated from N_2_-atomised LaserForm 17-4PH (B) powder. The concept is based on consecutive heat treatment procedures and diamond burnishing (DB) processes. A two-stage study was conducted. The first stage was an LPBF process experiment. The following combination of LPBF parameter values was selected after optimisation: a laser power of P=150 W, laser scanning speed of v = 1200 mm/s, and layer thickness of t=40 μm. In the second stage, this combination was used to evaluate the effects of two heat treatment procedures (HT1 and HT2) and two DB processes (using burnishing forces of 100 N and 300 N) on the tensile properties and surface integrity of LPBF 17-4PH SS cylindrical samples. The HT2 procedure, including annealing $(1200^{\circ }$, 4 h), solution treatment ($1060^{\circ }$, 1 h), cooling (${-70\;}^{\circ }C,2\;h$), and ageing ($482^{\circ }$, 4 h) led to yield limit, tensile strength, and Vickers hardness values of YL=1071 MPa, TS=1410 MPa, and 523 HV, respectively. The concept presented takes advantage of the combination of the transformation, precipitation and strain-hardening effects. The combined effect was most pronounced in the samples subjected to the HT2 procedure and subsequent DB (300 N), for which a retained austenite fraction of 6.93%, surface microhardness of ${563\;HV}_{0.05}$ and the maximum values of the compressive axial and hoop RSs of −1426.3 MPa and −1095.9 MPa, respectively, were measured.

## 1. Introduction

17-4PH is a precipitation-hardening martensitic stainless steel (SS) developed in the late 1940s to meet the demands of the rapidly developing aerospace industry [1]. This steel offers high strength and good toughness at elevated temperatures (up to ${316\;}^{\circ }C$), in combination with excellent corrosion resistance (comparable to AISI 304L austenitic SS), making it a preferred material for various applications in the marine, chemical, oil and gas, food, nuclear power, and medical industries [2,3,4,5,6]. The application of a range of standardised heat treatments to conventional 17-4PH SS (formed by hot rolling and drawing) provides a good balance of performance and cost efficiency [7].

Additive manufacturing (AM), also known as three-dimensional (3D) printing, is a perspective direction in the development of sustainable technologies. Over the past 30 years, AM has developed as a revolutionary, flexible technology for the industrial production of complex and efficient near-net-shape parts, with the ability to optimise processing time and minimise the number of components and material waste [8]. 17-4PH is typical of the SS used in various AM processes, according to the classification proposed by Kanishka and Acherjee [9]: metal material extrusion [10,11,12], direct energy deposition [4,13,14], selective laser melting (SLM), and direct metal laser sintering (DMLS) techniques [3,9]. The latter two processes belong to the category of laser powder bed fusion (LPBF) and are most widely used in metal AM technologies [1,15].

The focused energy of the laser beam during LPBF causes rapid solidification and high cooling rates, on the order of $10^{6}\;{}^{\circ}C\;s^{-1}$ [6], and complex thermo-physical phenomena within the powder bed and the molten melt pool and in the solidified phase, which occur at different spatial and temporal time scales [15]. Anisotropic heat conductivity during the melting and solidification processes causes a non-equilibrium, strongly anisotropic as-built microstructure containing mixed phases of δ-ferrite (BCC), austenite (FCC, γ), martensite (BCT, $\alpha^{\prime }$), and carbide precipitations [1]. This results in poor tensile [5,16,17,18,19] and fatigue [20,21] mechanical properties compared to conventional 17-4PH SS material.

The mechanical behaviour of as-built 17-4PH SS parts depends on the density, porosity distribution, and microstructural profile, which are dependent on the following main factors: (1) the laser energy density E, (2) the build direction (usually $0^{\circ }$ (horizontal) and $90^{\circ }$ (vertical), (3) the scanning strategy, and (4) the powder feedstock composition effect. For a specific part geometry and 3D printing system used, different combinations of the first three factors determine different thermal histories, while the fourth factor determines the material response according to a specific thermal history. Considering the specificity of precipitation-hardening SS and the unique microstructure of AM parts, the application of controlled post-manufacturing heat treatments has great potential for customising mechanical properties to suit the desired operational behaviour.

A limited number of studies have taken into account the influence of the laser energy density. Using two laser energy densities (53.12 and $73.4\;J/{mm}^{3}$), Lashgari et al. [22] established that a higher energy density leads to a higher volume fraction of retained austenite because of increased nitrogen absorption in SLM 17-4PH SS cylindrical samples. Pasebani et al. [23] investigated the effects of the atomising medium (nitrogen gas vs. water) and heat treatment on the microstructure and mechanical properties of 17-4PH SS for two energy density values (E = 64 and 104 J/${mm}^{3}$). These authors reported that a higher energy density improved the mechanical properties of heat-treated samples (solution treated at 1315 °C followed by ageing at 482 °C), with this effect being more pronounced for water-atomised powder.

Studies focused on the effect of build direction on the microstructure and mechanical properties of AM 17-4PH SS have not shown clear trends. Aripin et al. [2] compared the effects of $0^{\circ }$ and $90^{\circ }$ build directions in combination with heat treatments (H750 and H1100) on the microstructure and mechanical properties of SLM 17-4PH SS. These authors found that a $0^{\circ }$ build direction increased the hardness, martensite phase volume fraction, and yield and tensile strengths of as-built and heat-treated SLM 17-4PH samples. Chae et al. [5] showed that the tensile behaviours of as-built and heat-treated (direct ageing, solid solution, and solid solution followed by ageing) AM 17-4PH SS block samples were isotropic both parallel and perpendicular to the building direction. Lashgari et al. [24] investigated the effect of the scanning pattern (hexagonal and concentric), build orientation, and number of scans per layer on the microstructure, reciprocating dry sliding wear, and corrosion properties of AM 17-4PH SS and showed that the horizontal build direction led to a smaller volume fraction of retained austenite and higher hardness for both hexagonal and concentric scanning strategies. In addition, the hexagonal scanning strategy resulted in better wear resistance and corrosion resistance. Based on fully reversed (R = 1) strain-controlled fatigue tests, Yadollahi et al. [25] found that interlayer cavities/voids formed during SLM of vertically orientated samples were more detrimental than those for horizontally built samples as they caused greater stress concentration. Kim et al. [26] examined the effects of the build direction and various conventional heat treatments (combining a hot isostatic pressing, solid solution, and ageing treatment) on the porosity and mechanical properties of LPBF 17-4PH SS. The results showed that the horizontal build direction was preferable to the vertical one in terms of porosity, hardness, and yield strength, but at the expense of reduced plasticity.

The different standard heat treatment procedures [6] for conventional 17-4PH SS result in different mechanical properties because of their unique precipitation-hardening effects during ageing, by which they form sub-microscopic, Cu-rich phases within a martensitic matrix. The as-built and heat-treated mechanical properties of AM 17-4PH SS differ from those of the conventional material, even when the chemical composition and heat treatment are the same [5,6,14,20,26,27]. The reasons for this are the non-equilibrium as-built microstructure, with the inherent AM porosity resulting from rapid local heating and solidification, and the complex evolution of the microstructure during heat treatment. Studies have been carried out to define appropriate heat treatment procedures in terms of desired mechanical properties, with the temperature and duration of the solutionising and ageing treatments varied within relatively wide limits. Based on tensile test results, Chae et al. [5] optimised the ageing temperature of N_2_-atomised AM 17-4PH SS samples heat-treated in the range of 400–700 °C. These authors found that ageing above 490 °C worsened the yield strength and ductility and that a solutionising treatment at 1038 °C for 1 h, followed by ageing, improved strength and ductility, with tensile strengths reaching 1371–1399 MPa. Brown et al. [6] investigated the effects of varying the solutionising and homogenising temperatures (${930\;}^{\circ }C$, ${1040\;}^{\circ }C$, and ${1150\;}^{\circ }C$) on microstructure and in combination with standard ageing treatments (H900, H1025, and H1150) on the mechanical performance of Ar-atomised LPBF 17-4PH SS. For all solutionising/homogenising temperatures studied, the typical trend of change in the mechanical characteristics of the conventional analogue was observed: the yield strength, ultimate tensile strength, and ductility increased with decreasing ageing temperature, and a homogenisation treatment at ${1150\;}^{\circ }C$ resulted in improved strengths compared to a ${1040\;}^{\circ }C$ solution-treated material, albeit at the cost of reduced ductility. Lashgari et al. [28] evaluated the effects of solutionising at temperatures of ${1040\;}^{\circ }C$, ${1140\;}^{\circ }C$, and ${1240\;}^{\circ }C$ over 0.25 h to 4 h, with water quenching and ageing at a temperature of ${480\;}^{\circ }C$ from 1 h to 8 h, on the microstructure, volume fraction of retained austenite, hardness, and tensile properties of SLM 17-4PH SS. Solutionising at a temperature of ${1240\;}^{\circ }C$ for 2–4 h followed by ageing at ${480\;}^{\circ }C$ for 1–3 h minimised the amount of retained austenite (≈4.4%).

It is important to note that the as-built 17-4PH SS microstructure and the heat treatment response vary depending on the powder feedstock composition effect. The latter is primarily determined by the atomisation medium (N_2_-, Ar-, or water-atomised powder feedstocks) and to a lesser extent by the atmosphere used during the 3D printing (N_2_ or Ar). Meredith et al. [29] found that as-built AM 17-4PH SS fabricated using Ar- and N_2_-atomised feedstocks in ProX 200 (3D Systems, Rock Hill, United States) and EOS M280 (EOS GmbH, Munich, Germany) LPBF systems responded differently to standard heat treatment cycles, with changes in the response being strongly influenced by differences in the nitrogen content of the powder feedstock. AM samples fabricated from Ar-atomised feedstocks contain low levels of N_2_ (0.01 wt.%) and retained austenite (<1%) and respond to standard solutionising and ageing heat treatment similarly to conventional material. In contrast, AM specimens made from N_2_-atomised feedstocks contained between 0.06 and 0.12 wt.% N_2_ and up to 81% retained austenite, leading to a deviation from the expected over-ageing response with increasing ageing temperature. Based on tensile and rotating bending fatigue tests, Yin et al. [30] demonstrated the different mechanical responses of as-built LPBF AM 17-4PH SS samples fabricated from an N_2_-atomised powder feedstock versus five Ar-atomised feedstocks. The samples produced from the N_2_-atomised powder built in horizontal and vertical orientations showed the lowest yield strength but relatively high tensile strength, achieving a high fatigue strength in the high-cycle fatigue field because of the strain-induced transformation of austenite to martensite. Pasebani et al. [23] investigated the effects of atomising media and post-heat treatments using water-atomised and gas-atomised (in N_2_ media) 17-4PH SS powders. These authors reported that the gas-atomised powder revealed a single martensitic phase after 3D printing and heat treatment and that as-printed water-atomised powder contained martensitic and austenitic phases. Based on a detailed microstructural characterisation of SLM 17-4PH SS using three different Ar-atomised powders, Vunnam et al. [31] demonstrated an approach to achieving a fully martensitic 17-4PH component in the as-built condition by fine-tuning the alloy composition without heat treatments. The authors governed the phase transformation from δ-ferrite to austenite (γ) and subsequently to martensite (α′) by varying the concentrations of ferrite and austenite stabilising elements, as represented by a chromium-to-nickel equivalent (${Cr}_{eq}/{Ni}_{eq}$) value. Using two Ar-atomised powder feedstocks, Sabooni et al. [32] established that the as-built microstructure could be almost fully martensitic or ferritic, depending on the ${Cr}_{eq}/{Ni}_{eq}$ ratio of the feedstock powder, and that increasing the ${Cr}_{eq}/{Ni}_{eq}$ ratio leads to a higher fraction of δ-ferrite phase in the as-built condition. Therefore, the atomising media and gas environment used in the AM process are important factors in selecting appropriate post-heat treatment procedures. For N_2_-atomised AM 17-4PH SS, Cheruvathur et al. [16] presented a specific heat treatment procedure involving homogenising annealing at 1150 °C for more than 90 min, followed by solutionising at 1050 °C, followed by quenching in an agitated brine solution to achieve a uniform reproducible microstructure (containing approximately 90% martensite and 10% austenite) and increase the microhardness to that expected for a conventional material. Emphasising the effect of using N_2_ as an atomisation medium, Lass et al. [17] developed an alternative post-build thermal processing protocol consisting of homogenising at ${1150\;}^{\circ }C$ for 1 h, quenching in room-temperature water, solutionising at 1050 °C for 1 h, quenching in room-temperature water, cooling to ${-40\;}^{\circ }C$, and holding for 30 min. The resulting microstructure contains 95% martensite and about 5% retained austenite and has a yield strength exceeding 90% of that of conventional material heat-treated according to Condition A (ASTM A564) [33] (solutionising at ${1040\;}^{\circ }C$ for 1 h). Yeon et al. [34] proposed a post-heat treatment procedure for LPBF 17-4PH SS fabricated under N_2_ conditions consisting of annealing (${1200\;}^{\circ }C$ for 4 h + furnace cooling), solutionising (${1060\;}^{\circ }C$ for 1 h + gas cooling), and ageing (${482\;}^{\circ }C$ for 4 h + air cooling). A yield strength of 1264 MPa and elongation of 12.9% were obtained.

Applying heat treatments to as-built AM 17-4PH SS is the most effective way to improve the bulk properties of fabricated components. Considering the notably poor surface quality of as-built AM parts and the importance of surface integrity (SI) for the operational behaviour and functionality of rotary metal elements, the surface post-treatment processes play a key role. Therefore, an effective approach to improving the quality and operational behaviour of AM 17-4PH SS parts is the development of combined post-processing technologies, including appropriate heat treatments and surface treatment techniques. According to Maleki et al. [35], surface post-treatments applied to AM metallic materials fall into the following categories: material removal, no material removal, coatings, and hybrid treatments.

From the perspective of sustainable manufacturing, the surface cold working processes as part of no-material-removal surface post-treatment processes are of interest. The surface cold working processes are based on severe surface plastic deformation, which produces a significant strain-hardening effect in the surface layers, introduces beneficial compressive residual stresses, and changes the surface texture. In other words, surface cold working modifies the complex state of the surface and subsurface layers, significantly improving the SI [36]. Surface cold working processes are dynamic (implemented through impact/impulse action of the deforming elements) or static (burnishing).

Applications of both dynamic and static surface cold working processes in post-processing procedures of AM parts are described in the literature. Using three types of peening media (steel shot, glass, and ceramic beads), Swietlicki et al. [37] demonstrated that shot peening of DMLS 17-4PH SS caused microstructure refinement and induced $\alpha^{\prime }$-martensite formation, increasing the hardness and corrosion resistance. Walczak et al. [38] reported that shot peening of AM 17-4PH SS increased the coefficient of friction by 15.5% and 20.7%, while the wear factor decreased by 25.9% and 32.7% for the samples peened with CrNi steel shot and ceramic beads, respectively. Based on plane bending-fatigue tests, Tsuchiya et al. [39] evaluated the effect of laser shock peening on the fatigue strength of solution heat-treated AM maraging steel without and with crack-like surface defects (semicircular slits). The laser peening increased the fatigue limit (at $10^{7}$ cycles) of a smooth specimen and a specimen with a 0.2 mm deep semicircular slit by 43% and 114%, respectively, due to the introduced significant compressive residual stresses. Travieso-Disotuar et al. [40] demonstrated the effectiveness of combining processes, including milling and vibration-assisted ball burnishing, to optimise the surface roughness and mechanical properties of SLM maraging steel components. Zhang et al. [41] applied the ultrasonic nanocrystal surface modification technique (using a sliding impacting tip) to improve the fatigue behaviour of the SLM AISI 316 SS.

The advantages of burnishing processes over dynamic processes are the achievement of a remarkable smoothing effect combined with a surface hardening effect and the introduction of useful residual compressive stresses. Thus, burnishing processes are particularly suitable for finishing rotary components such as shafts, axles and guides. The above effects are sensitive to the type of tangential contact between the deforming element (roller or ball) and the treated surface, i.e., rolling (roller or ball burnishing) or sliding (slide burnishing) friction contact. Yaman et al. [42] reported that heat treating followed by roller burnishing led to an increase in the wear resistance of LPBF AM Inconel 718 parts of 55%. For comparison, the wear resistance increased by 38% when the roller burnishing process was applied to an as-built part. Brock et al. [43] showed that hydrostatic ball burnishing of LPBF AISI 316L SS cylindrical specimens reduced the roughness from Ra=5.57 μm to Ra=0.201 μm and transformed the tensile residual stresses after LPBF into significant compressive residual stresses (axial and hoop stresses of −694 MPa and −333.5 MPa, respectively). Kebede et al. [44] investigated successive grinding and slide diamond burnishing of AM MetcoAdd 17-4PH-A SS blocks with a focus on the effect of the burnishing parameters on the surface roughness, residual stress, and microhardness. The combination of grinding and diamond burnishing (DB) yielded a significant improvement in terms of SI.

However, information is lacking on the effectiveness of combined post-processing processes consisting of sequential heat treatments and burnishing of AM 17-4PH SS parts. Such combined processes, which have significant technological potential for achieving a synergistic effect because of precipitation hardening and strain hardening, are of scientific and practical interest for significantly improving the quality and performance of AM 17-4PH SS rotary parts. Thus, the main objective of the present study was to evaluate the effects of sequential heat treatment procedures and DB processes on the tensile properties and SI of LPBF 17-4PH SS cylindrical specimens, fabricated from N2-atomised LaserForm 17-4PH (B) powder feedstock.

## 2. Materials and Methods

### 2.1. 17-4PH SS Powder

**Figure 1:**
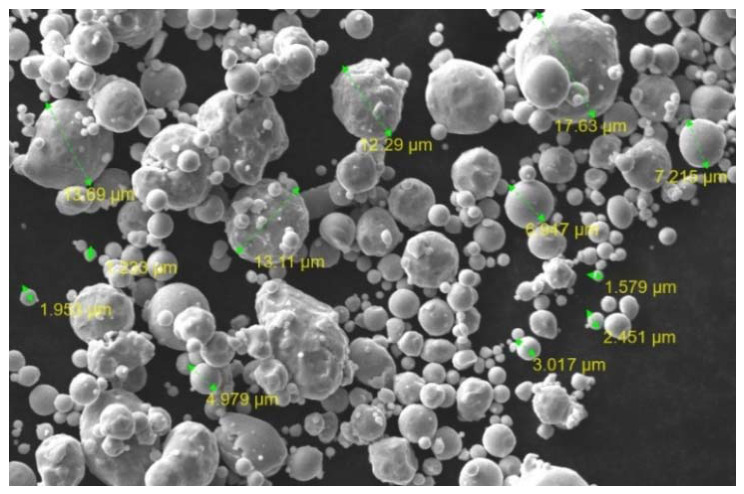
SEM image of 17-4PH (B) SS powder.

Nitrogen-atomised LaserForm 17-4PH (B) SS powders (obtained from 3D-Systems, Inc., Rock Hill, SC, USA) were used in this study. Figure 1 shows the powder morphology, with spherical shapes ranging in size from 1.23 to 17.63 μm. Despite the relatively large size range, small particle sizes dominate. The average size was approximately 7–8 μm.

The chemical composition was established using a Zeiss Evo 10 (Carl Zeiss Microscopy GmbH, Jena, Germany) scanning electron microscope (SEM) and energy-dispersive (EDS) X-ray analysis (Table 1). The nominal chemical composition of the 17-4PH (B) stainless steel powder used complies with ASTM Standard A693-16 [45] for 17-4PH stainless steel.

### 2.2. LPBF Process Experiment

An LPBF process experiment was conducted to select parameter values that would provide an as-built microstructure with optimal mechanical, physical, and metallurgical properties. Prismatic samples of 17-4PH SS were fabricated in an N_2_ atmosphere using a ProX300 DMP 200 Type B machine (3D Systems Inc., Riom, France). All samples were printed in the $0^{\circ }$ build direction (horizontally on the build plate). The experimental design is shown in Table 2.

The laser energy density E, which changes for each experimental point, was determined using Equation (1):

(1) $$E=\frac{P}{v\;t\;h},J/{mm}^{3},$$

where h=const=0.06 mm is the hatch spacing.

**Figure 2:**
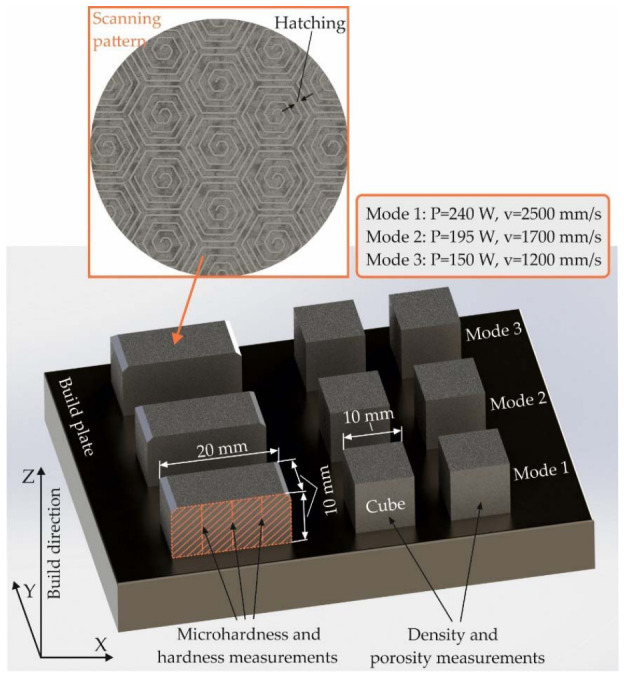
LPBF experiment specimens.

For each of the three studied layer thickness values (t=30, 40, and 50 μm), three series of samples were fabricated using three modes (Figure 2). These modes were defined by different combinations of laser power and laser scanning speed according to Table 2. These two parameters together have a determining effect on the thermal history of the as-built structure. The higher the laser power is, the higher the laser scanning speed is. The scanning pattern shown in Figure 2 was used with a layer rotation of $60^{\circ }$. Each series consisted of one prismatic and two cubic samples. Samples were removed from the build plate by wire EDM AV35, (ONA Electroerosión, S.A., Durango, Spain) and then ground to form the final dimensions (JE600, BLOHM JUNG GmbH, Hamburg, Germany).

The prismatic samples were used to measure the Vickers hardness HV, microhardness ${HV}_{0.05}$, volume fraction of retained austenite, and microstructure in the as-built state. The samples were mounted in a conductive copper-filled resin, sequentially ground with diamond discs (with grades from 80 to 2400), polished in two steps using a diamond slurry (with 3 μm and then 1 μm particles), and finally chemically etched with a 3% nitric acid solution.

The Vickers hardness was determined using a VEB-WPM tester (WPMWerkstoffprüfsysteme Leipzig GmbH, Markkleeberg, Germany) with a 10 kg load and a 10 s holding time. Three measurements were recorded and arithmetically averaged. A ZHVμ microhardness tester (Zwick/Roell, Ulm, Germany) was used to measure the microhardness using a load of 0.05 kgf and a holding time of 10 s. The median of the clustering of 30 measurements (10 measurements along three lines in the build direction, as shown in Figure 2) of a sample was taken as the final surface microhardness value. The microstructures were observed using SEM. DIFFRAC.DQuant V1.5, developed by Bruker (Billerica, MA, USA), was used to measure the retained austenite volume fraction.

The two cubic samples from each series were used to determine the average values of the measured density and porosity. The mass of the samples was measured via an analytical balance (Kern ADB 200-4; Kern & Sohn GmbH, Balingen, Germany). An Ultrapyc 5000 gas pycnometer (Anton Paar GmbH, Graz, Austria) was used to determine the density $D\;(g/{cm}^{3})$ and porosity P (%) by determining the exact volume of a sample through nitrogen gas displacement.

Thus, the optimal combination of LPBF parameter values was selected based on the analysis of six material characteristics of as-built structures, ranked according to their priority in the following sequence: the microstructure, volume fraction of retained austenite, Vickers hardness HV, microhardness ${HV}_{0.05}$, density D, and porosity P. Using the selected combination of optimal LPBF parameter values, cylindrical samples were printed to study the effects of sequential post-heat treatment procedures and the DB process on their mechanical properties and SI characteristics.

### 2.3. Post-Heat Treatment Procedures

The post-heat treatment procedures applied in this study were tailored to the specifics of the N_2_-atomised LaserForm 17-4PH (B) SS powder feedstocks and N_2_ gas used during the LPBF process. Previous research [29,30] has shown that as-built specimens fabricated from N_2_-atomised feedstocks contain between 0.06 and 0.12 wt.% N_2_, which causes a significantly higher volume fraction of retained austenite (up to 81%). The two post-heat treatment procedures used to minimise this effect are shown in Table 3. The first heat treatment procedure (HT1) was presented by Yeon et al. [33] for LPBF 17-4PH SS parts fabricated under N_2_ conditions. The second heat treatment procedure (HT2) is based on the idea of maximising the martensite volume fraction by including an additional cooling treatment before the ageing treatment (Table 3). The inclusion of a cooling treatment is of key importance given the expected significant amount of retained austenite in the as-built structure due to the N_2_-atomised powder feedstocks used and the N_2_ atmosphere used during the LPBF process. The selected cooling temperature (${-70\;}^{\circ }C$) favours reaching the end point of the martensitic transformation, stimulating the formation of a more homogeneous structure dominated by martensite. This favours the formation of Cu-rich precipitates in the martensite matrix during the subsequent ageing process. In addition, maximising the martensite volume fraction reduces internal stresses, stabilising the dimensions of the fabricated parts.

**Figure 3:**
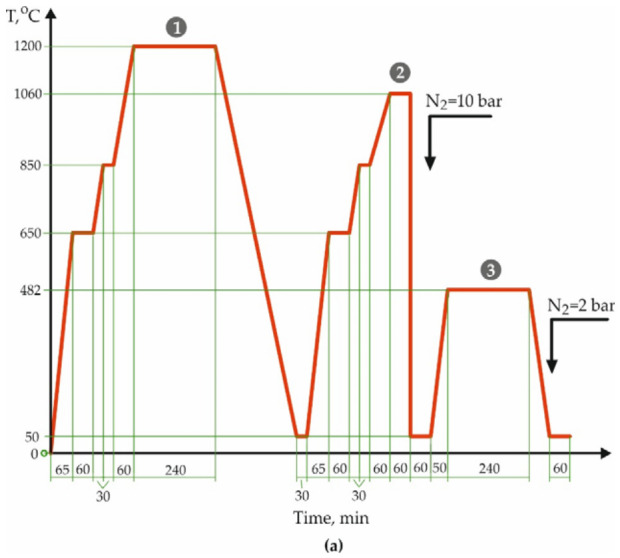

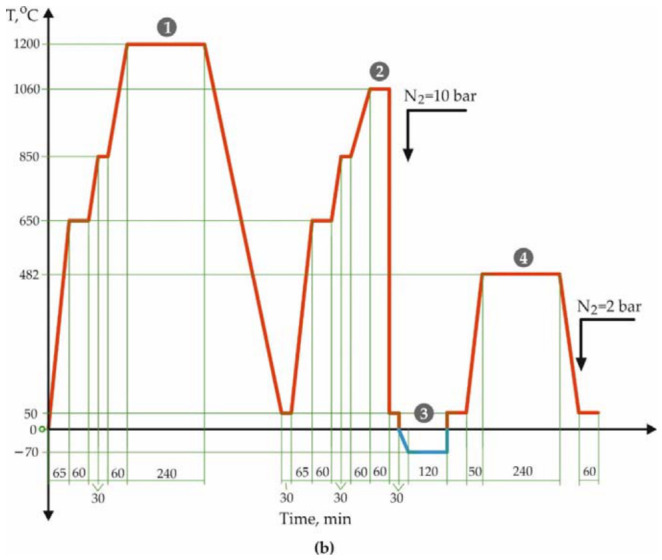
Time–temperature curves: (**a**) HT1 and (**b**) HT2.

Figure 3a,b show the time–temperature curves for HT1 and HT2.

Both heat treatments were performed on the as-built cylindrical samples using a vacuum furnace HTS-245 (HTS Vacuum Furnaces S.r.l., Mozzanica, Italy) with a control thermocouple to ensure that the target temperature was maintained within $\pm {2\;}^{\circ }C$. The cooling treatment was carried out using an ICT C750 cold chamber (DBS Cooling Technology (Suzhou) Co., Ltd., Suzhou, China).

### 2.4. Turning and Diamond Burnishing

Machining of as-built samples includes preliminary and fine turning operations. DB was used as the finishing process, as it is particularly effective for treating cylindrical parts [46]. Turning and DB were implemented on a TM2500 turning–milling CNC processing centre (RAIS, Pazardzhik, Bulgaria) in one clamping process. A HELIL 2525-3T20 holder with a GRIP 3015Y IC807 cutting insert (ISCAR Ltd., Migdal Tefen, Israel) was used in the turning process for tensile test samples. A VCMT 160404-F3P cutting insert and an SVVCN 2525M–16 holder (ISCAR Ltd., Migdal Tefen, Israel) were used in the turning process for cylindrical samples. The fine turning parameters were as follows: cutting velocity of 100 m/min, feed rate of 0.1 mm/rev, and cutting depth of 0.5 mm.

**Figure 4:**
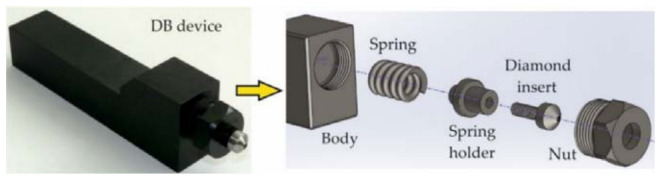
DB device.

DB was conducted under flood lubrication conditions using the burnishing device, providing elastic normal contact between the spherical-ended polycrystalline deforming diamond insert and the treated surface (Figure 4). Two DB force magnitudes were used: Fb=100 and 300 N. The remaining DB governing factors were kept constant as follows: a diamond insert radius of 3 mm, sliding velocity of 70 m/min, and feed rate of 0.05 mm/rev.

### 2.5. Study of the Effects of Sequential Post-Heat Treatment Procedures and DB Processes

**Figure 5:**
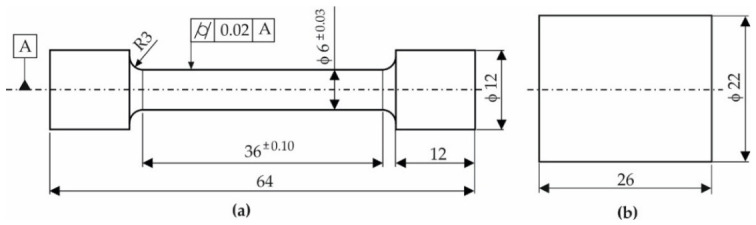
Geometry of the samples for (**a**) the uniaxial tensile test, and (**b**) SI measurements.

The present study aimed at evaluating the effects of sequential post-heat treatment procedures and DB processes on the tensile properties and evolution of SI in LPBF 17-4PH SS cylindrical specimens. Using the selected optimal LPBF parameter values, two types of cylindrical samples were printed, intended respectively for uniaxial tensile tests and for evaluating the effects of post-heat treatment procedures and DB finishing processes on SI characteristics. Figure 5 shows the final geometry of the samples after machining (previous and fine turning).

Table 4 shows the specifications of the types of cylindrical specimens, including sample designations, preparation conditions, and detailed information about the measured mechanical and SI characteristics.

A Zwick/Roell Vibrophore 100 testing machine (Ulm, Germany) was used for tensile tests at room temperature. The mechanical properties of the tensile test specimens (AB, HT1, and HT2) were determined as arithmetic mean values obtained from eight samples each.

The Ra roughness parameter was measured using a Mitutoyo Surftest SJ-210 surface roughness tester (Mitutoyo Corporation, Kawasaki, Japan). Arithmetic mean values were obtained from measurements performed on six equally spaced sample generatrices.

The Vickers hardness was determined as the arithmetic mean value of six measurements on the front surface of each of the cylindrical samples in accordance with the test parameters described in Section 2.2. The surface microhardness ${HV}_{0.05}$ was determined as the arithmetic mean value of twelve measurements on the cylindrical surfaces of the samples. To assess the microhardness profile in-depth, microstructure cross-sectional surface samples were prepared from each group of cylindrical specimens. The procedure for preparing each samples consisted of the following steps: (1) mounting the samples in a specially made metal clamp; (2) grinding the sample using diamond discs with grits of 80, 220, 600, 1200, and 2400; (3) polishing the sample with suspensions containing abrasive particles with sizes of 3 μm and 1 μm; and (4) chemical etching with aqua regia containing nitric acid and hydrochloric acid at a ratio of 1:3. The microhardness profile ${HV}_{0.05}$ was arithmetically averaged from 22 measurements in three radial directions, oriented 120° apart. The microstructure was observed via Zeiss Evo 10 scanning electron microscopy.

A Bruker D8 ADVANCE diffractometer and the DIFFRAC.EVA V5.2 software (Billerica, MA, USA) were used to analyse the presenting phases. A Bruker D8 Advance diffractometer with a pin-hole collimator with a primary beam with a 1 mm diameter was used to measure the axial and hoop residual stresses (RSs). The X-ray Cr tube’s mode of operation (voltage/current) was 30 kV/40 mA. The sin^2^ψ method was used with a least squares fitting procedure to evaluate the axial and hoop RSs. Electropolishing was used to remove layers sequentially while determining the RS distribution. A Kristall 650 device (QATM, Mammelzen, Germany) was used (room temperature of 20–22 °C; current 0.9 A). The electrolyte used contains ethanol and perchloric acid in a ratio of 10:1. The duration of the process varies depending on the thickness of the layer to be removed: from 20 s to remove a layer about 1–2 μm thick near the surface to 120 s to remove a layer about 10 μm thick at a greater depth.

### 2.6. Flow-Chart of the Study

**Figure 6:**
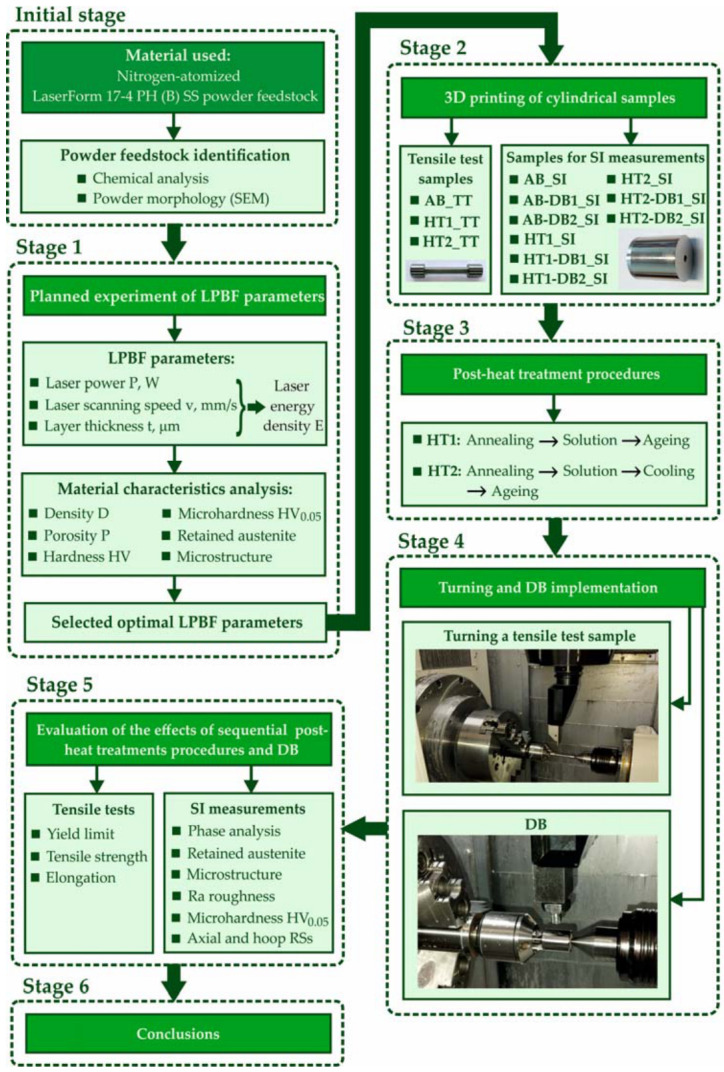
Flow-chart.

Figure 6 shows a flow-chart of the study.

## 3. Results and Discussion

### 3.1. Selection of an Optimal Combination of LPBF Parameters

**Figure 7:**
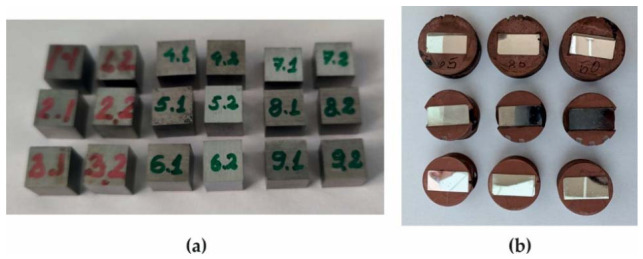
Samples for material characteristics measurements: (**a**) density and porosity; (**b**) Vickers hardness, microhardness, retained austenite, and microstructure.

The material characteristics of the as-built parts, which varied depending on the LPBF parameter values, were measured using the prismatic and square specimens fabricated according to the LPBF experimental design process (Figure 7).

The density D of the as-built parts varied from 7.77 to $7.82\;g/{cm}^{3}$, except for a value of $7.715\;g/{cm}^{3}$ obtained for the fourth experimental point (Table 5). No clear trend for density or porosity as a function of the laser energy density was observed. The density of LPBF parts depends on many factors that determine the density of powder bed, including the particle shape, particle size, powder surface morphology, and flowability of the powder [47]. The powder flowability directly determines the uniform distribution of the powder and hence the variations in density and porosity in different regions. The powder used in this study was characterised by small particle sizes (average size of 7–8 μm) with spherical shapes (Figure 1), which improved the powder flowability. As a result, the density obtained was higher than the measured density of $7.7\;g/{cm}^{3}$ of LPBF 17-4PH SS parts fabricated from gas-atomised powders with an average particle size of 13 μm after solution treatment (at 1051 °C) and ageing (at 482 °C) [23]. In fact, the porosity measurement method used registers the presence of pores only in the surface areas of the samples, which explains the significant scatter of the measured porosity P, % (Table 5).

**Figure 8:**
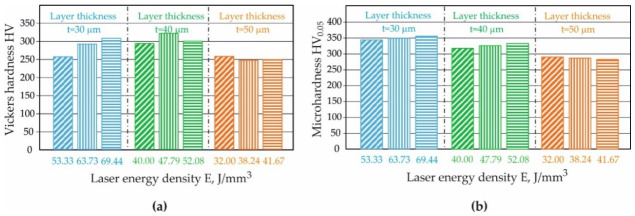
Effects of LPBF process parameters on (**a**) Vickers hardness HV and (**b**) microhardness ${HV}_{0.05}$.

Figure 8a,b illustrate the effects of the LPBF process parameters on the Vickers hardness HV and microhardness ${HV}_{0.05}$. The layer thickness has a significant influence on the hardness and microhardness. The LPBF implementation with the greatest layer thickness studied (t=50 μm) minimises both of these mechanical characteristics because of the greater heterogeneity of the resulting as-built structures. In general, the laser energy density has a greater influence on hardness, with this effect being most pronounced at the smallest layer thickness of 30 μm (Figure 8a). The LPBF implementation with a laser power of P=195 W, laser scanning speed of v=1700 mm/s, and layer thickness of t=40 μm resulted in the maximum Vickers hardness of 321 HV. The following microhardness trends were observed: with increasing layer thickness, the microhardness decreased; increased laser energy density at fixed layer thicknesses of t=30 μm and 40 μm increased the microhardness; and this latter trend reversed at a layer thickness of t=50 μm (Figure 8b).

**Figure 9:**
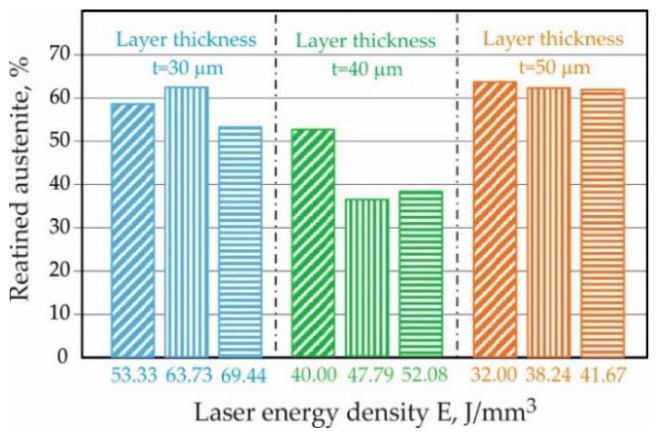
Effect of LPBF process parameters on the volume fraction of retained austenite.

Figure 9 illustrates the effect of the LPBF process parameters on the volume fraction of retained austenite. A correlation exists between the measured values of Vickers hardness (Figure 8a) and the volume fraction of retained austenite: samples with less retained austenite had higher Vickers hardness values because austenite is a softer phase. The highest Vickers hardness of 321 HV and the lowest volume fraction of retained austenite were measured in the specimen printed with LPBF parameter values corresponding to experimental point No. 5 (Table 5).

**Figure 10:**
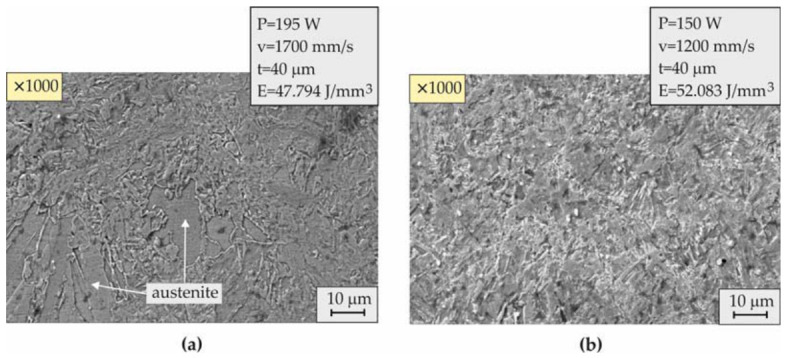
Microstructures of the samples (**a**) printed with LPBF parameters according to experimental point No. 5 and (**b**) printed with LPBF parameters according to experimental point No. 6.

To identify the optimal combination of LPBF process parameter values, it was necessary to compare the microstructures of the samples (Figure 10a,b) to determine the microstructure for which the lowest values of the volume fraction of retained austenite were measured, along with relatively high values of the Vickers hardness and microhardness ${HV}_{0.05}$ (Figure 8a,b).

The microstructure of the sample printed with LPBF parameter values corresponding to experimental point No. 5 was significantly more heterogeneous because it contained zones with large austenite grains (Figure 10a). Based on the results, the optimal combination of LPBF process parameter values was selected (Table 6).

### 3.2. Tensile Test Results

**Figure 11:**
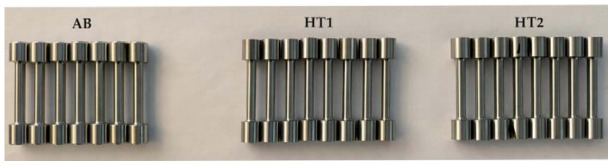
Tensile test samples.

Figure 11 shows the three groups of tensile test specimens.

**Figure 12:**
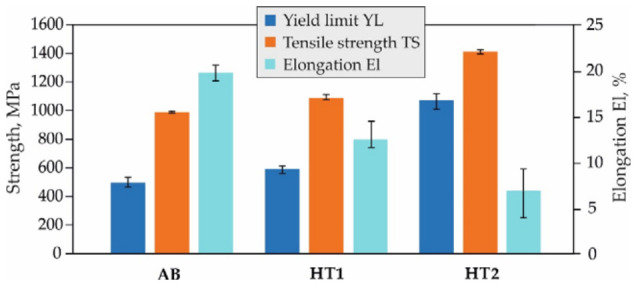
Mechanical characteristics.

The values determined for the mechanical characteristics (yield limit YL, tensile strength TS, and elongation El) clearly demonstrate the effectiveness of the heat treatment procedures (Figure 12). The HT1 procedure achieved a yield limit and tensile strength of YL=592 MPa and TS=1087 MPa, respectively, which are 19.58% and 10.12% higher, respectively, than the analogous mechanical characteristics for AB samples. The HT2 procedure maximised the yield limit and tensile strength, which reached YL=1071 MPa and TS=1410 MPa, respectively. These values are 116.22% and 42.75% higher than the corresponding mechanical characteristics in the as-built state, which confirms the significant effect of adding the cooling treatment in HT2. As expected, the trends of increasing yield limit and tensile strength occur at the expense of decreasing the elongation El of the HT1 and HT2 samples (Figure 12).

A comparison of the mechanical characteristics obtained with those reported by Yeon et al. [33], who presented the HT1 heat treatment procedure for LPBF samples using N_2_-atomised 17-4PH SS powders obtained from 3D-Systems, is of interest. The yield limit and tensile strength of the HT1 samples in the present study were significantly lower than those reported by Yeon et al. [34], but the elongations were practically the same (El=12.457% in the present study versus 12.95% [34]). However, the tensile strength (TS=1410.851 MPa) obtained after HT2 exceeded that reported by Yeon et al. [34]. In addition, the HT2 procedure achieved a tensile strength exceeding that of AM 17-4PH SS printed in the horizontal build direction and conventionally wrought 17-4PH SS after solutionising and ageing [5] and was very close to the maximum tensile strength of 1496 MPa obtained after homogenising (${1150\;}^{\circ }C,\;1\;h)$ and H900 ageing of LPBF 17-4PH SS using Ar-atomised feedstocks [6].

A one-way analysis of variance was conducted to assess the significance of heat treatment on the measured mechanical properties (yield limit YL, tensile strength TS and elongation El) using the QstatLab 5.0 software [48]. The heat treatment factor was varied at three levels as follows: AB, HT1, and HT2. Eight observations were made for each level. The selected significance level was α=0.05. Table 7 summarises the results obtained. The null hypothesis is rejected because the calculated *p*-values (0.00) are less than the significance level (0.05). Therefore, the heat treatment factor is concluded to be significant.

**Figure 13:**
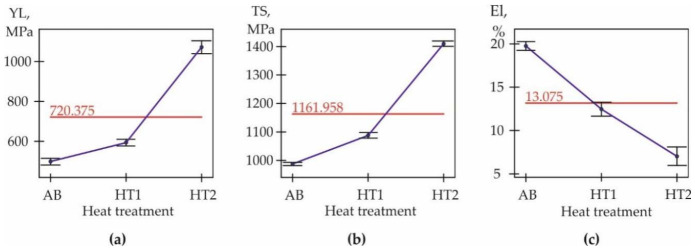
Main effects and confidence intervals: (**a**) YL, (**b**) TS, and (**c**) El.

Figure 13 illustrates the main effects and confidence intervals for the mean values (individual residual standard deviations) corresponding to the factor levels. The red lines show the overall averages. The absence of a horizontal line that can intersect all confidence intervals is evidence of the significance of the heat treatment factor.

### 3.3. Effects of Sequential Heat Treatment Procedures and DB Processes on SI

#### 3.3.1. Phase Analysis Results

**Figure 14:**
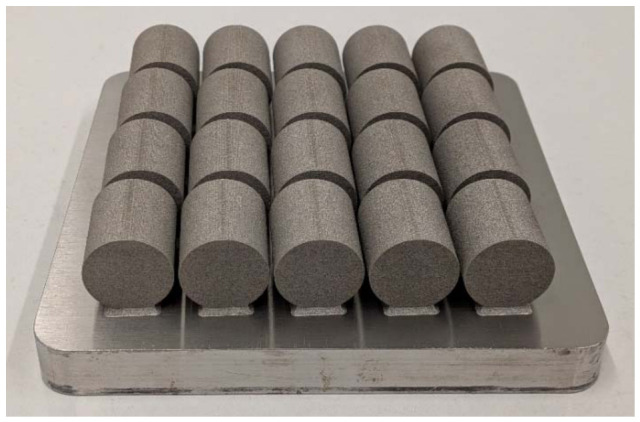
As-built samples for SI measurements.

The as-built samples used for SI measurements (Figure 14) were distributed into nine groups and processed according to the preparation conditions specified in Table 4.

**Figure 15:**
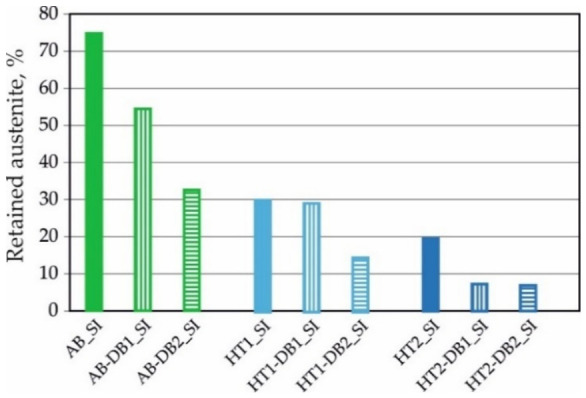
Effects of heat treatment procedures and DB processes on the retained austenite.

Figure 15 shows the effects of sequential heat treatment procedures and DB processes on the retained austenite in the samples’ surfaces. Significantly more retained austenite was observed in the AB_SI samples (74.79%) than in the prismatic sample (38%) corresponding to the optimal combination of LPBF parameter values (Table 5). The reasons for the stabilisation of a larger amount of austenite are the higher temperature in the interior and the lower cooling rate due to the significantly larger overall dimensions of the cylindrical specimens. For comparison, the volume of the cylindrical samples ($9883.45\;{mm}^{3}$) is 4.94 times larger than the volume of the prismatic samples. The implementation of the LPBF process with the same parameters provides practically the same thermal impact on the individual microvolumes of both types of samples, but during the layer-by-layer construction, more heat accumulates in the larger cross-sections of the cylindrical samples and crystallisation is slowed down. This can lead to microsegregation of nickel and copper in the non-equilibrium structure, which locally lowers the initiation temperature of the martensitic transformation, expanding the austenitic region.

The HT1 and HT2 procedures reduced the retained austenite by 59.64% and 73.84%, respectively, compared to as-built structures. Subsequent DB processes provoke the austenite → α′-martensite phase transformation in all three structures. In other words, the inherent DB strain-hardening effect leads to a transformation surface hardening effect, i.e., deformation martensite is induced in the surface layers. Therefore, as a result of the sequential heat treatment procedures and DB processes, the following three hardening effects occur: (1) transformation and precipitation-hardening effects through the full volume of the samples due to HT1 and HT2, (2) a strain-hardening effect in the surface layer due to DB, and (3) austenite → α′-martensite transformation surface hardening due to DB. This synergistic effect was most pronounced in the samples from groups HT2-DB1_SI and HT2-DB2_SI, in which the retained austenite proporitons were 7.33% and 6.93%, respectively (Figure 15). The effect of the strain-induced transformation of austenite into α′-martensite was established by Swietlicki et al. [37] in DMLS 17-4PH SS cylindrical samples after shot peening.

**Figure 16:**
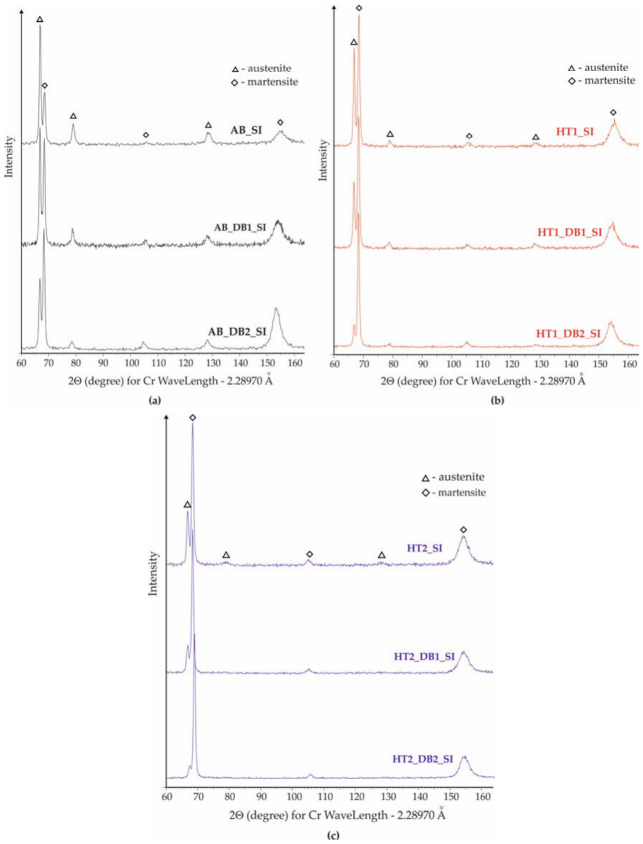
Phase analysis depending on heat treatment procedures and DB processes: (**a**) as-built state, (**b**) heat-treated according to HT1, and (**c**) heat-treated according to HT2.

The phase analysis clearly shows the effects of the heat treatment procedures and DB processes (Figure 16a–c). A significant change was observed in the ratio of the peak intensities of the austenitic to martensite phase in the doublet peaks γ (111) and α (110) because of the following effects: (1) transformation and precipitation-hardening effects due to HT1 and HT2 procedures and (2) austenite → α′-martensite phase transformation in the surface layers due to DB processes. The effect of DB on the austenite → α′-martensite phase transformation is most pronounced in AB-DB1_SI and AB-DB2_SI (Figure 16a) due to the higher austenite volume fraction (Figure 15). Figure 16b shows a trend of decreasing austenite peak intensities in samples HT1-DB1_SI and HT1-DB2_SI compared to sample HT1_SI in correlation with the magnitude of the deforming force used in the DB process. The diffractogram of sample HT2-DB2_SI clearly shows the absence of austenite peaks γ (200) and γ (220) (Figure 16c) due to the combined effects of HT2 and DB.

#### 3.3.2. Hardness and Microhardness

**Figure 17:**
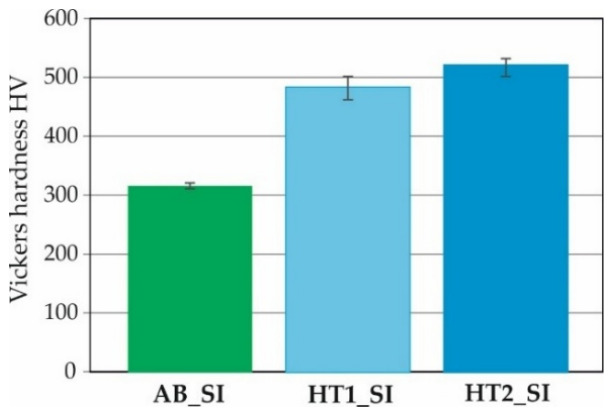
Vickers hardness depending on the heat treatment procedure.

Figure 17 shows the average Vickers hardness values obtained for the three studied structures. HT1 and HT2 provide Vickers hardnesses of 485 HV and 523 HV, which is an increase of 52.88% and 64.98%, respectively, compared to the as-built state (AB_SI samples). An additional advantage of the HT2 procedure compared to HT1 is less scatter, which is an indicator of the more homogeneous structure obtained as a result of the higher volume fraction of martensite and the formation of coherent, copper-rich precipitates in the martensite matrix. The average Vickers hardness value of 317 HV for AB_SI samples (Figure 17) is twice as high as that of approximately HV=156 reported by Kim et al. [26] for as-built 17-4PH SS samples printed in the horizontal build direction.

**Figure 18:**
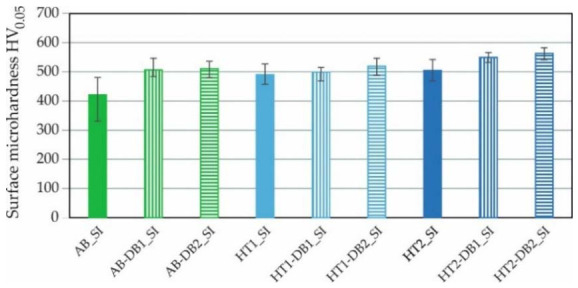
Effects of heat treatment procedures and DB processes on the surface microhardness ${HV}_{0.05}$.

Figure 18 illustrates the effects of the HT1 and HT2 heat treatment procedures and DB processes on the surface microhardness ${HV}_{0.05}$. The surface microhardness of the turned specimens (AB_SI, HT1_SI, and HT2_SI) follows the established trend for the Vickers hardness (Figure 17). For the three structures studied, DB increased the surface microhardness in proportion to the magnitude of the burnishing force. As expected, the HT2 treatment followed by DB with a burnishing force of 300 N maximised the above-described combination of hardening effects, leading to the highest surface microhardness value of ${563\;HV}_{0.05}$ for the specimens in group HT2-DB2_SI (Figure 18).

**Figure 19:**
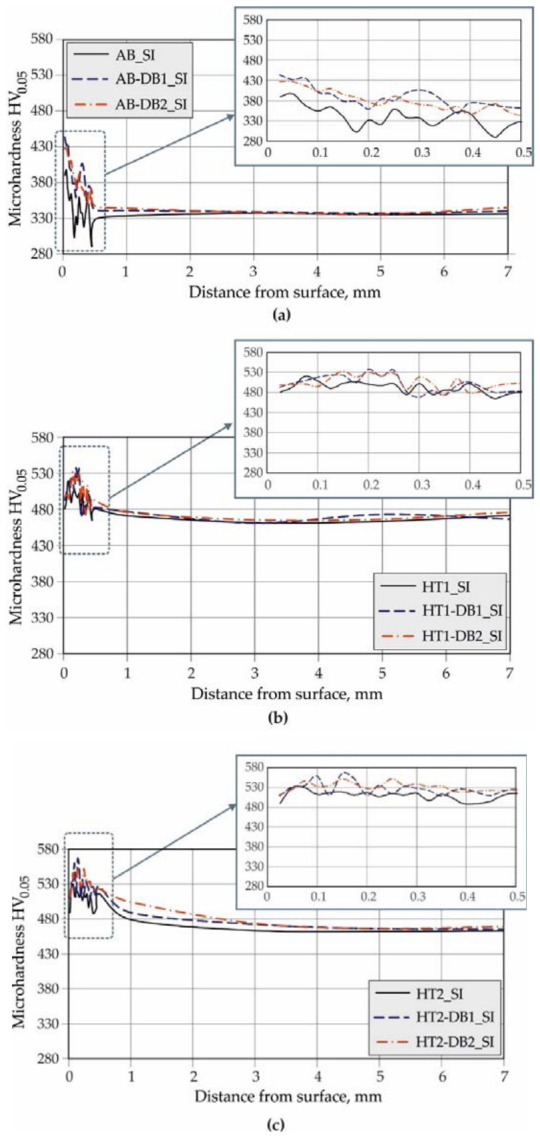
Effects of heat treatment procedures and DB processes on microhardness profiles ${HV}_{0.05}$ in-depth. (**a**) AB_SI group specimens; (**b**) HT1_SI group specimens and (**c**) HT2_SI group specimens.

Figure 19a–c illustrate the microhardness profiles ${HV}_{0.05}$ for the three material states of the cylindrical specimens (AB_SI, HT1_SI, and HT2_SI) after turning and DB processes with the two studied magnitudes of the deforming force (100 N and 300 N). Fluctuations in the microhardness profile near the surface, up to 0.5 mm, were observed in all specimens, with the profiles significantly differing in qualitative and quantitative aspects for the three material states. The variations in microhardness were related to the presence of pores, the phase, and the microstructural inhomogeneity. The most pronounced fluctuations in the turned specimens of the AB_SI group (Figure 19a) indicate greater phase and microstructural inhomogeneity in the as-built state.

In quantitative terms, the microhardness profiles of all groups of samples near the surface are consistent with the trends observed for the surface microhardness (Figure 18): (1) DB provoked strain hardening in all three structures, but this effect was most pronounced in the as-built state because of the amount of retained austenite (Figure 15); (2) the HT2 treatment maximised the measured values of ${HV}_{0.05}$ (Figure 19c), followed by the HT1 treatment (Figure 19b). To a certain extent, DB processes increased the thickness of the gradient-affected zone. This effect was most noticeable in samples HT2-DB1_SI and HT2-DB1_SI, in which the thickness of the gradient-affected layer is approximately 3 mm (Figure 19c).

#### 3.3.3. Roughness

**Figure 20:**
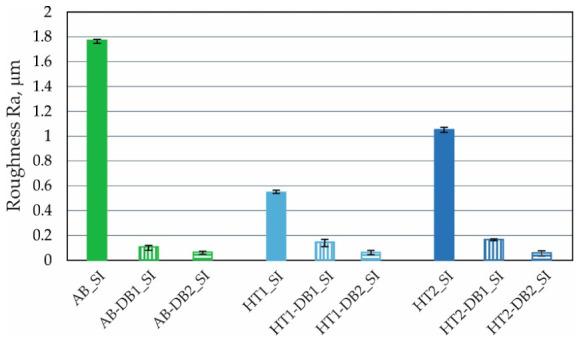
Effects of heat treatment procedures and DB processes on the $R_{a}$ roughness parameter.

The average roughness of the cylindrical surfaces after 3D printing was $R_{a}=15.397\;\mu m$. Turning and DB processes drastically reduced the $R_{a}$ of the cylindrical surfaces for the three studied structures (as-built, HT1, and HT2) (Figure 20).

The resulting roughness after turning was lower in the specimens subjected to HT1 ($R_{a}=0.546\;\mu m$) than in the specimens subjected to HT2 ($R_{a}=1.051\;\mu m$). The smoothing effect of DB with a burnishing force of 100 N was most pronounced in the AB-DB1_SI samples ($R_{a}=0.143\;\mu m$), followed by the HT1-DB1_SI samples ($R_{a}=0.143\;\mu m$), and least pronounced in the HT2-DB1_SI samples ($R_{a}=0.166\;\mu m$) (Figure 20). These results show that the roughness decreased with increasing plasticity, i.e., a correlation was observed between $R_{a}$ and the Vickers hardness of the material for the three material states (Figure 17). Applying DB with three times the deforming force (300 N) resulted in mirror surfaces in the three groups of samples, with very close roughness values of approximately $R_{a}=0.06\;\mu m$.

#### 3.3.4. Residual Stress

**Figure 21:**
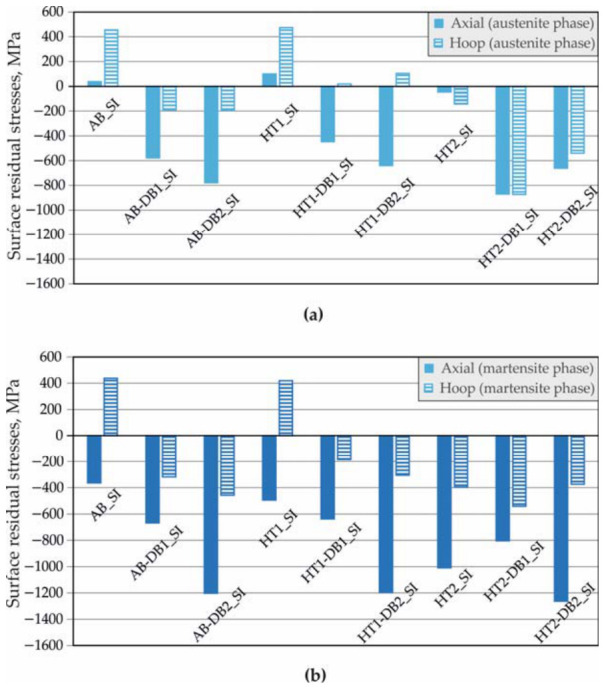
Effects of heat treatment procedures and DB processes on surface residual stress for (**a**) austenite phase and (**b**) martensite phase.

Figure 21 shows the surface axial and hoop RSs in the nine groups of cylindrical specimens, determined for the austenitic and martensitic phases. The errors of the measured RSs are shown in Table 8. There is a correlation between the reported errors and the ratio between the volume fractions of the two phases. The larger the errors were in the RSs reported for the respective phase, the smaller the volume fraction of that phase was in the respective specimen. As expected, relatively large errors were observed for the axial and hoop RSs, measured as follows: for the martensitic phase in specimens AB_SI and AB-DB1_SI; for the austenitic phase in specimens HT1-DB2_SI, HT2_SI, HT2-DB1_SI, and HT2-DB2_SI (Table 8). These results are consistent with those obtained for the measured retained austenite in the groups of cylindrical specimens (Figure 15).

The surface RSs measured for the martensite phase (Figure 21b) were higher than those for the austenite phase (Figure 21a) because of volumetric expansion and the higher hardness and strength of the martensite. In all three studied structures, DB processes introduced significant compressive surface axial and hoop RSs in the martensite phase, as well as compressive surface axial RSs in the austenite phase. In contrast to the turned samples (AB_SI, HT1_SI, and HT2_SI), both types of surface RSs in the martensite phase were completely compressive in all samples subjected to DB (Figure 21b). The reasons for this are as follows. The HT1 and HT2 heat treatment procedures provoke the austenite → martensite phase transformation, and the subsequent DB process causes the austenite → α′-martensite strain-induced transformation. These effects are accompanied by a volumetric expansion of the martensite crystals and a high dislocation density in the surface layers of the samples. As a result, significant surface compressive stresses are formed. As expected, the maximum surface compressive axial residual stress of −1263 MPa was measured for the martensite phase in the sample HT2-DB2_SI subjected to the HT2 procedure and DB with a burnishing force of 300 N (Figure 21b).

**Figure 22:**
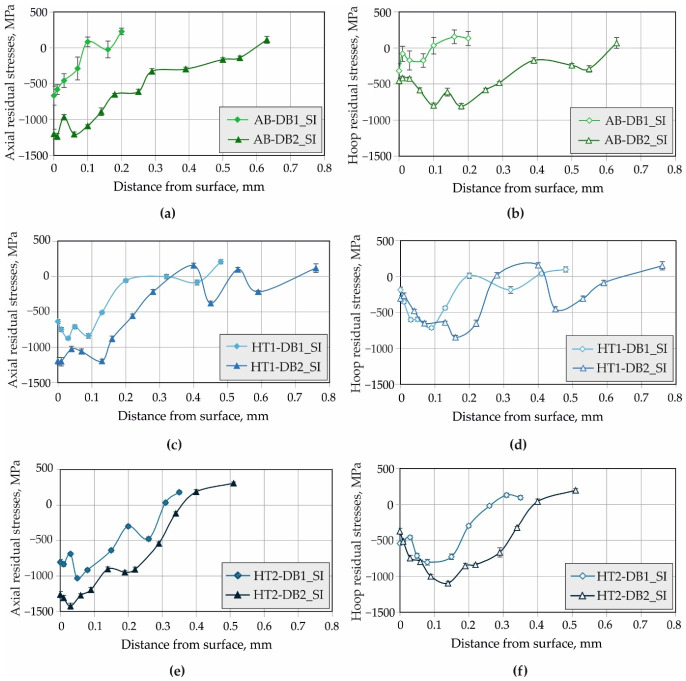
Profiles of axial and hoop RSs: (**a**) axial RS in as-built state, (**b**) hoop RS in as-built state, (**c**) axial RS in heat-treated state according to HT1, (**d**) hoop RS in heat-treated state according to HT1, (**e**) axial RS in heat-treated state according to HT2, and (**f**) hoop RS in heat-treated state according to HT2.

Figure 22a–f shows the profiles of axial and hoop RSs depending on heat treatment procedures and DB processes, calculated for the martensite phase. DB processes introduce a zone of useful axial and hoop compressive RSs in the surface layers of all specimens, but the absolute values of RSs and the depth of the compressive zone change depending on the material structure (as-built, HT1, and HT2).

The graphs in Figure 22 show the following trends: (1) The axial RSs are larger in absolute value than the hoop RSs in all three material structures, which indicates that the strain-hardening effect dominates over the transformation hardening effect [49]. (2) The increase in burnishing force (300 N) led to an increase in the absolute value of the introduced compressive axial and hoop RSs and the depth of the compression zone due to the greater amount of strain-induced $\alpha^{\prime }$-martensite and the greater dislocation density in the surface layers. (3) In general, the maximum absolute values in the profiles of both types of RSs increased with increasing hardness of the material structure before DB; the maximum values of the compressive axial and hoop RSs of −1426.3 MPa and −1095.9 MPa, respectively, were observed for specimen HT2-DB2_SI, whose structure was characterised by the highest Vickers hardness of 523 HV (Figure 17) and the lowest amount of retained austenite (Figure 15). The results obtained for RSs cannot be compared with others, since there are no publications in the literature dedicated to DB or other cold working processes of AM 17-4PH SS cylindrical parts.

#### 3.3.5. Microstructure

The objects of microstructural analysis were the turned samples (AB_SI, HT1_SI, and HT2_SI) and the samples subjected to the DB process with a burnishing force of 300 N (AB-DB2_SI, HT1-DB2_SI, and HT2-DB2_SI), which were representatives of the three structures of the material.

**Figure 23:**
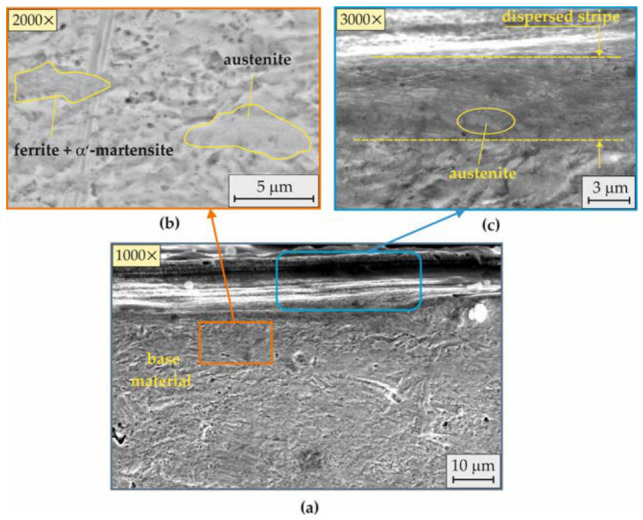
Microstructure of AB_SI sample: (**a**) magnification 1000×; (**b**) magnification 2000×; (**c**) magnification 3000×.

The microstructure of the turned specimen in the as-built state (AB_SI) is heterogeneous in terms of morphology and phase composition (Figure 23). The structure of the base material contains a significant amount of retained austenite (more than 70%, attributable to the effect of geometry on the thermal history) (Figure 15), ferrite, and martensite (Figure 23b). The phase composition favours the formation of a layer on the cutting insert, which causes a rougher surface (Figure 23a,c). When removing chips, a deformation stripe is formed in the surface layer, which turns into a dispersed stripe (Figure 23c) due to the phase transformation austenite → $\alpha^{\prime }$-martensite and the increased dislocation density. Fine-grained austenite is also observed in the dispersed zone.

**Figure 24:**
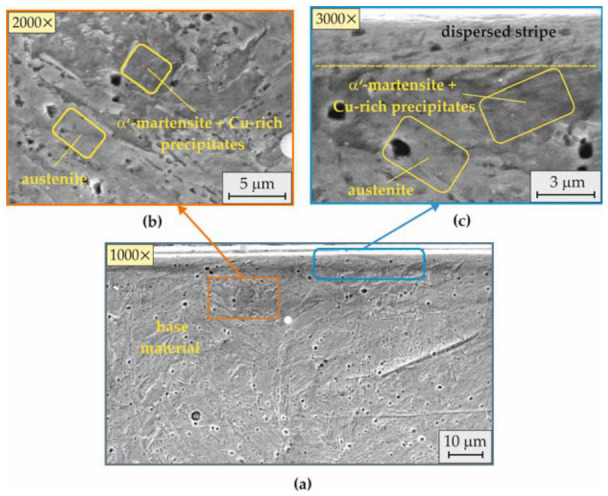
Microstructure of HT1_SI sample: (**a**) magnification 1000×; (**b**) magnification 2000×; (**c**) magnification 3000×.

Figure 24a shows the microstructure of the turned sample HT1_SI, heat-treated according to HT1. The heat treatment significantly homogenises the structure due to the phase transformation austenite → martensite; as a result, the amount of retained austenite decreases to 30% (Figure 15). Dispersed precipitates of Cu-rich phases within the resulting martensite are observed due to ageing treatment (Figure 24b). The higher hardness of the material favours the formation of a significantly smoother surface (Figure 20). As a result, a narrower dispersed stripe is formed (Figure 24c). The transition of the base material to the dispersed stripe passes through a deformation stripe. In addition to dispersed martensite, sub-dispersed precipitates of Cu-rich phases are observed in the dispersed zone, which are a product of dynamic ageing (due to the deforming impact of the cutting insert).

**Figure 25:**
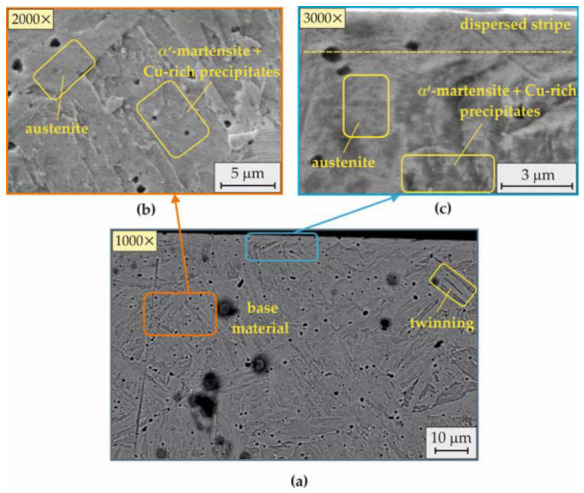
Microstructure of HT2_SI sample: (**a**) magnification 1000×; (**b**) magnification 2000×; (**c**) magnification 3000×.

Figure 25a shows the microstructure of the turned sample HT2_SI, subjected to the HT2 procedure.

Morphologically, the structure has the form of intersecting stripes as a result of the cooling treatment, which causes additional stresses due to contraction of the material. These stresses provoke a twinning mechanism, additional decomposition of austenite into martensite, and dispersed precipitates of Cu-rich phases (Figure 25b,c). As a result of the cooling treatment, the retained austenite is reduced to 19.56% (Figure 15). The increased amount of martensite and dispersed precipitates results in a significantly harder structure (Vickers hardness of 523 HV (Figure 17)). The small amount of austenite is transformed into $\alpha^{\prime }$-martensite as a result of the mechanical impact of the cutting tool on the surface layer. The significant amount of saturated martensite is characterised by a highly difficult sliding mechanism and is only dispersed in the zone of cutting insert impact. As a result, the dispersed stripe formed is relatively narrower (Figure 25c).

**Figure 26:**
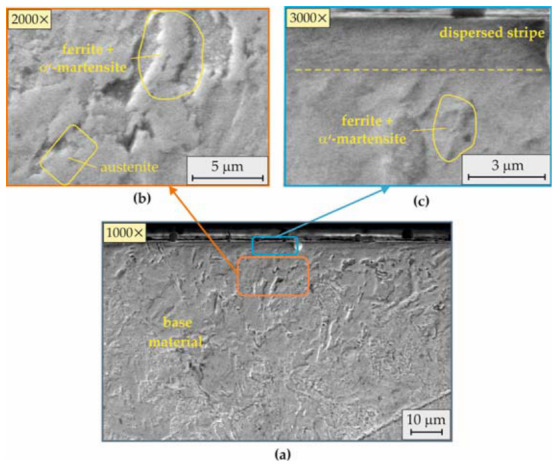
Microstructure of AB-DB2_SI sample: (**a**) magnification 1000×; (**b**) magnification 2000×; (**c**) magnification 3000×.

Figure 26a shows an integral picture of the microstructure in the as-built state after DB with a burnishing force of 300 N (AB-DB2_SI sample). The structure of the base material (Figure 26b) is similar to that of the AB_SI sample (Figure 23a), with the difference being mainly in the surface layer. The deforming impact due to the DB process provokes the following effects in the surface zone (Figure 26c): (1) a smoothing effect, which leads to a smooth cylindrical surface; (2) an austenite → $\alpha^{\prime }$-martensite phase transformation in the dispersed stripe; and (3) dispersion of the retained austenite.

**Figure 27:**
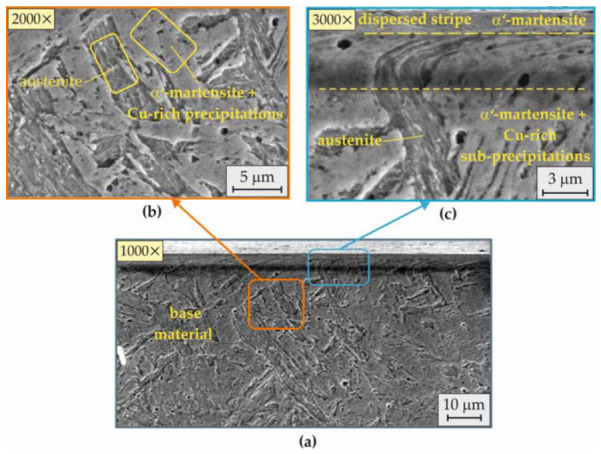
Microstructure of HT1-DB2_SI sample: (**a**) magnification 1000×; (**b**) magnification 2000×; (**c**) magnification 3000×.

Figure 27a shows an integral picture of the microstructure of sample HT1-DB2_SI, which was subjected to the HT1 heat treatment procedure and DB with a burnishing force of 300 N. The structure of the base material (Figure 27b) is similar to the structure of sample HT1_SI (Figure 24a), with the difference being in the surface layer due to the deforming effect of the DB process. The surface layer is smoothed and deformed in the direction of burnishing velocity (Figure 27a). Two zones are observed in the surface layer (Figure 27c): (1) a zone with a pronounced deformation band, including martensite, tangentially deformed austenite grains, dispersed austenite and dispersed precipitates of Cu-rich phases; (2) a narrow dispersed stripe, located immediately near the surface, containing dispersed martensite and sub-dispersed Cu-rich precipitates, which are a product of dynamic ageing (caused by the deforming impact of the diamond insert).

**Figure 28:**
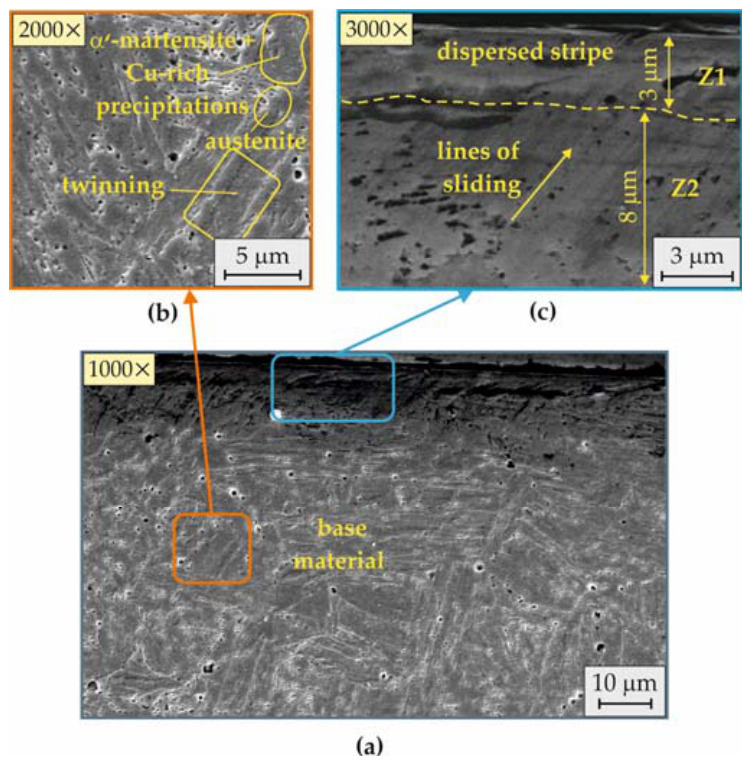
Microstructure of HT2-DB2_SI sample: (**a**) magnification 1000×; (**b**) magnification 2000×; (**c**) magnification 3000×.

The microstructure of the HT2-DB2_SI sample (Figure 28) illustrates the effects from the HT2 heat treatment procedure with respect to the base material (Figure 28b) and the subsequent DB (300 N) with respect to the surface layer.

Two zones are observed in the surface layer (Figure 28c): zone Z1, reaching the surface, and zone Z2, bordering the base material. Pronounced deformation stripes and slip lines are observed in zone Z2. In this zone, part of the retained austenite has undergone an austenite → $\alpha^{\prime }$-martensite phase transformation, resulting in the retained austenite reaching the lowest recorded amount of 6.93% (Figure 15) and the remaining amount of the austenite being highly dispersed. The outer zone Z1 is a narrow dispersed zone containing mainly martensite and sub-dispersed Cu-rich precipitates that is hardly visible at the magnification considered.

## 4. Conclusions

A new combined post-processing concept for improving the quality of LPBF 17-4PH SS cylindrical parts fabricated from N_2_-atomised LaserForm 17-4PH (B) powder feedstock was presented. The concept is based on consecutive heat treatment procedures and DB processes. Using the selected optimal combination of LPBF parameter values, the effects of two heat treatment procedures (HT1 and HT2) and two DB processes (using burnishing forces of 100 N and 300 N) on the tensile properties and SI of LPBF 17-4PH SS cylindrical samples were evaluated. The major new findings of this research are as follows:

➢ Based on a designed experiment, the following optimal combination of LPBF parameter values was selected with respect to N_2_-atomised LaserForm 17-4PH (B) powder feedstock: laser power P=150 W, laser scanning speed v=1200 mm/s, and layer thickness t=40 μm.
➢ The HT2 heat treatment procedure, including annealing $(1200^{\circ }$, 4 h), solution ($1060^{\circ }$, 1 h), cooling (${-70\;}^{\circ }C,2\;h$) and ageing ($482^{\circ }$, 4 h), leads to a yield limit, tensile strength, and Vickers hardness of YL=1071 MPa, TS=1410 MPa and 523 HV, respectively. These values are 116.22%, 42.82%, and 64.98% higher, respectively, than the analogous mechanical characteristics in the as-built state and are due to the resulting more homogeneous microstructure.
➢ The post-processing concept, including sequential HT1 and HT2 heat treatment procedures and DB processes, achieves the following three hardening effects in LPBF 17-4PH SS cylindrical parts: (1) transformation and precipitation-hardening effects in the full volume due to HT1 and HT2, (2) a strain-hardening effect in the surface layer due to DB, and (3) austenite → α′-martensite transformation surface hardening due to DB. This combination of effects is most pronounced in the samples subjected to the HT2 heat treatment procedure and subsequent DB process with a burnishing force of 300 N, as indicated by the retained austenite of 6.93% and surface microhardness of ${563\;HV}_{0.05}.$
➢ Turning and DB processes drastically reduced the value of the $R_{a}$ roughness parameter of the cylindrical surfaces. This effect was more pronounced in the samples subjected to HT1 and HT2. After turning, the lowest roughness value of $R_{a}=0.546\;\mu m$ was obtained for the samples subjected to HT1, followed by the samples subjected to HT2 ($R_{a}=1.051\;\mu m$). DB with a burnishing force of 300 N had a pronounced smoothing effect regardless of the material condition (as-built, HT1, or HT2), i.e., mirror-smooth surfaces with values of approximately $R_{a}=0.06\;\mu m$.
➢ DB processes create a zone of useful axial and hoop compressive RSs in the surface layers of LPBF 17-4PH SS cylindrical samples. The maximum absolute values of both types of RSs increase with increasing material hardness. The maximum values of the compressive axial and hoop RSs of −1426.3 MPa and −1095.9 MPa, respectively, were recorded in the specimen subjected to the HT2 procedure and subsequent DB process with a burnishing force of 300 N.
➢ The presented combined post-processing concept provides effective improvement of the quality of LPBF 17-4PH SS rotary components. It is of scientific and practical interest to evaluate the technological capabilities of this concept from the point of view of the scale factor of the manufactured rotary components, which will be the subject of our next work.

## Figures and Tables

**Table 1:** Chemical composition of the 17-4PH (B) SS powder.

Elements	Specified Ranges (wt%)	EDS (wt%)
Fe	Balance	72.35
Cr	15.0–17.5	17.23
Ni	3.0–5.0	3.77
Cu	3.0–5.0	3.59
Si	≤1.00	0.59
Mn	≤1.00	0.89
Nb	0.15–0.45	0.2
Mo	-	0.21
Co	-	0.09
C	≤0.07	-

**Table 2:** Experimental design of LPBF process.

№	Laser Power P, W	Laser Scanning Speed v mm/s	Layer Thickness t, μm	Laser Energy Density E, J/mm^3^
1	240	2500	30	53.333
2	195	1700	30	63.725
3	150	1200	30	69.444
4	240	2500	40	40
5	195	1700	40	47.794
6	150	1200	40	52.083
7	240	2500	50	32
8	195	1700	50	38.235
9	150	1200	50	41.666

**Table 3:** Post-heat treatment procedures used.

№	Designation	Heat Treatment Conditions
1	HT1	(1) Annealing ($1200^{\circ }$, 4 h + Furnace cooling) → (2) Solution ($1060^{\circ }$, 1 h + Gas cooling) → (3) Ageing ($482^{\circ }$, 4 h + Air cooling)
2	HT2	(1) Annealing ($1200^{\circ }$, 4 h + Furnace cooling) → (2) Solution ($1060^{\circ }$, 1 h + Gas cooling) → (3) Cooling (${-70\;}^{\circ }C,2\;h$) → (4) Ageing ($482^{\circ }$, 4 h + Air cooling)

**Table 4:** Sample specifications.

Type	Designation	Preparation Condition	Measurements
Tensile test samples	AB	As-built → Machining (turning + fine turning)	Yield limit YL, MPa; Tensile strength TS, MPa; Elongation El, %.
	HT1	As-built → HT1 → Machining	
	HT2	As-built → HT2 → Machining	
Samples for SI measurements	AB_SI	As-built → Machining (turning + fine turning)	Phase analysis; Retained austenite; Microstructure; Vickers hardness; R_a_ roughness parameter; Surface microhardness ${\mathrm{HV}}_{0.05}$; Microhardness profile ${\mathrm{HV}}_{0.05}$ in-depth; Surface axial and hoop residual stresses (RSs).
	AB-DB1_SI	As-built → Machining → DB, Fb = 100 N	Phase analysis; Retained austenite; Ra roughness parameter; Microstructure; Surface microhardness ${\mathrm{HV}}_{0.05}$; Microhardness profile ${\mathrm{HV}}_{0.05}$ in-depth; Surface axial and hoop RSs; Profiles of axial and hoop RSs, MPa.
	AB-DB2_SI	As-built → Machining → DB, Fb=300 N	
	HT1_SI	As-built → HT1 → Machining	Phase analysis; Retained austenite; Microstructure; Vickers hardness; R_a_ roughness parameter; Surface microhardness ${\mathrm{HV}}_{0.05}$; Microhardness profile ${\mathrm{HV}}_{0.05}$ in-depth; Surface axial and hoop RSs.
	HT1-DB1_SI	As-built → HT1 → Machining → DB, Fb = 100 N	Phase analysis; Retained austenite; R_a_ roughness parameter; Microstructure; Surface microhardness ${\mathrm{HV}}_{0.05}$; Microhardness profile ${\mathrm{HV}}_{0.05}$ in-depth; Surface axial and hoop RSs; Profiles of axial and hoop RSs, MPa.
	HT1-DB2_SI	As-built → HT1 → Machining → DB, Fb = 300 N	
	HT2_SI	As-built → HT2 → Machining	Phase analysis; Retained austenite; Microstructure; Vickers hardness; R_a_ roughness parameter; Surface microhardness ${\mathrm{HV}}_{0.05}$; Microhardness profile ${\mathrm{HV}}_{0.05}$ in-depth; Surface axial and hoop RSs.
	HT2-DB1_SI	As-built → HT2 → Machining → DB, Fb = 100 N	Phase analysis; Retained austenite; R_a_ roughness parameter; Microstructure; Surface microhardness ${\mathrm{HV}}_{0.05}$; Microhardness profile ${\mathrm{HV}}_{0.05}$ in-depth; Surface axial and hoop RSs; Profiles of axial and hoop RSs, MPa.
	HT2-DB2_SI	As-built → HT2 → Machining → DB, Fb = 300 N	

**Table 5:** Measured material characteristics of the as-built parts depending on the LPBF parameters.

No.	Laser Power P, W	Laser Scanning Speed v, mm/s	Layer Thickness t, μm	Laser Energy Density E, J/mm^3^	Density D, g/cm^3^	Porosity P, %	Vickers Hardness HV	Micro-Hardness HV_0.05_	Retained Austenite %
1	240	2500	30	53.333	7.8	0.04	256	343	58.35
2	195	1700	30	63.725	7.785	0.04	291	348	62.25
3	150	1200	30	69.444	7.805	0.02	307	355	53.00
4	240	2500	40	40	7.715	0.01	293	318	52.30
5	195	1700	40	47.794	7.775	0.05	321	326	36.15
6	150	1200	40	52.083	7.775	0.04	299	333	38.00
7	240	2500	50	32	7.775	0.095	257	290	63.45
8	195	1700	50	38.235	7.82	0.045	246	286	62.07
9	150	1200	50	41.666	7.82	0.04	248	282	61.69

**Table 6:** Selected optimal combination of LPBF process parameters.

Laser Power P, W	Laser Scanning Speed v, mm/s	Layer Thickness t, μm
150	1200	40

**Table 7:** Computed one-way ANOVA results on the significance of the heat treatment.

Mechanical Characteristic	Source	Sum of Squares	Degrees of Freedom	Dispersion	F-Value	*p*-Value
Yield limit, MPa	HT, x1	1,512,217.000	2	756,108.500	1024.496	0.000
	Residual	15,498.625	21	738.029		
	Total	1,527,715.625	23			
	Residual standard deviation = 27.1667; R-sq = 0.98986; R-sq (adj) = 0.98889					
Tensile strength, MPa	HT, x1	778,840.583	2	389,420.291	4321.461	0.000
	Residual	1892.375	21	90.113		
	Total	780,732.958	23			
	Residual standard deviation = 9.492; R-sq = 0.99758; R-sq (adj) = 0.99735					
Elongation, %	HT, x1	649.8475	2	324.92375	335.50824	0.000
	Residual	20.3375	21	0.96845		
	Total	670.1850	23			
	Residual standard deviation = 0.98410; R-sq = 0.96965; R-sq (adj) = 0.96676					

**Table 8:** Surface axial and hoop RS errors for austenite and martensite phase.

Samples	Error Axial Austenite Phase	Error Hoop Austenite Phase	Error Axial Martensite Phase	Error Hoop Martensite Phase
AB_SI	65.2	128.9	63.5	54.6
AB-DB1_SI	46.4	41.7	132.6	99.4
AB-DB2_SI	83.6	86.6	68.2	42.3
HT1_SI	97.8	47.1	42.1	59.7
HT1-DB1_SI	56.9	43.9	26.5	38.3
HT1-DB2_SI	248.2	162.2	49.8	43.3
HT2_SI	280.5	92.5	37.1	33
HT2-DB1_SI	230.6	168.4	37.7	28.8
HT2-DB2_SI	443.6	211.7	44.6	43

## Data Availability

The original contributions presented in the study are included in the article; further inquiries can be directed to the corresponding author.
